# Nutritional and Functional Roles of Phytase and Xylanase Enhancing the Intestinal Health and Growth of Nursery Pigs and Broiler Chickens

**DOI:** 10.3390/ani12233322

**Published:** 2022-11-28

**Authors:** Vitor Hugo C. Moita, Sung Woo Kim

**Affiliations:** Department of Animal Science, North Carolina State University, Raleigh, NC 27695, USA

**Keywords:** broiler chickens, functional roles, intestinal health, nursery pigs, 3-phytase, 6-phytase, xylanase

## Abstract

**Simple Summary:**

Feed enzymes have been widely used with the goal to improve nutrient digestibility and growth performance of pigs. However, recent studies have shown that feed enzymes, especially phytase and xylanase, may provide potential benefits associated with the intestinal health and microbiota of nursery pigs and broiler chickens. Phytase and xylanase are catalyzers for the hydrolysis of phytic acid and β-1,4- xylan bonds, respectively. With a reduction in the antinutritional properties of phytic acid and non-starch polysaccharides (NSP) by the supplementation of these enzymes, there may be a possibility to improve the intestinal health of nursery pigs and broiler chickens. Intestinal health can be a determinant for the overall health and subsequent performance of the animals. Some of the factors affecting the intestinal health of nursery pigs could be related to antinutritional properties from phytic acid and NSP. Thus, this review paper aimed to discuss the nutritional and functional roles associated phytase and xylanase supplementation enhancing the intestinal health and growth of nursery pigs and broiler chickens.

**Abstract:**

This review paper discussed the nutritional and functional roles of phytase and xylanase enhancing the intestinal and growth of nursery pigs and broiler chickens. There are different feed enzymes that are currently supplemented to feeds for nursery pigs and broiler chickens. Phytase and xylanase have been extensively studied showing consistent results especially related to enhancement of nutrient digestibility and growth performance of nursery pigs and broiler chickens. Findings from recent studies raise the hypothesis that phytase and xylanase could play functional roles beyond increasing nutrient digestibility, but also enhancing the intestinal health and positively modulating the intestinal microbiota of nursery pigs and broiler chickens. In conclusion, the supplementation of phytase and xylanase for nursery pigs and broiler chickens reaffirmed the benefits related to enhancement of nutrient digestibility and growth performance, whilst also playing functional roles benefiting the intestinal microbiota and reducing the intestinal oxidative damages. As a result, it could contribute to a reduction in the feed costs by allowing the use of a wider range of feedstuffs without compromising the optimal performance of the animals, as well as the environmental concerns associated with a poor hydrolysis of antinutritional factors present in the diets for swine and poultry.

## 1. Introduction

Intestinal health is one of the most discussed contemporary issues in animal nutrition, due to its significance in the overall biological response of the animals. It is usually described as based on a combination of different parameters from different metabolic and physiological reactions that can impact the overall growth and health [1]. According to the literature, some of those parameters could be the quantification of anti and proinflammatory cytokines, immunoglobulins, and oxidative damage products in the digestive tract; assessment of intestinal morphology; and relative abundance and diversity quantification of the mucosa-associated microbiota in the jejunum [1,2,3,4,5,6,7].

In the first weeks after weaning and hatching, nursery pigs and broiler chickens start consuming diets with a greater amount of plant-based feedstuffs, which can contain different antinutritional factors and allergenic compounds that can lead to negative impacts on nutrient digestibility, growth performance, and intestinal health [8,9,10]. Corn and soybean meal are the most commonly used plant-based feedstuffs in diets for nursery pigs and broiler chickens, which can contain antinutritional and allergic compounds, such as phytic acid, non-starch polysaccharides (NSP), glycinin, and β-conglycinin [11,12]. The negative effects associated with phytic acid and NSP altering the digestion process may also lead to negative impacts in the intestinal health and intestinal microbiota of the animals, such as increases in the oxidative stress and increases in the abundance of pathogenic bacteria, which can be determinant for the intestinal health and subsequent growth of animals [5,13,14,15].

Enzymes are organic catalysts that accelerate reactions and act on specific substrates and reagents [16,17]. The enzyme activity can be affected by different factors, such as feed mixing temperature, levels of targeted substrate in the feedstuffs, different levels, types, and combinations of enzymes, and optimal gastric and intestinal pH and temperature [16,18]. The use of feed enzymes has been notarized as an alternative to increase nutrient digestibility and growth performance of swine and poultry through the active hydrolysis of antinutritional factors and allergenic compounds present in their diets [19,20]. Monogastric animals, especially pigs, at their first stages of growth, cannot effectively secrete endogenous enzymes, and consequently have their nutrient digestibility and utilization affected [6,21]. Recent studies have shown feed enzymes, especially phytase and xylanase, might play functional roles on intestinal health of nursery pigs and broiler chickens, whilst still providing the benefits on nutrient digestibility and growth performance [5,13,22,23].

Phytase is a feed enzyme that catalyzes the hydrolysis of phytic acid increasing the bioavailability of nutrients, especially phosphorus (P) and consequently leading to benefits on nutrient digestibility and growth performance [24,25]. Changes in the bioavailability of nutrients, such as calcium (Ca) and phosphorus, may express different effects on the intestinal and bone health, and composition and diversity of the intestinal microbiota of nursery pigs and broiler chickens [14,26,27,28]. The supplementation of xylanase will catalyze the depolymerization of the xylan structure into shorter chains and to the breaking down of the cell wall matrix [29,30]. Changes in the chemical structure of xylan and in the physicochemical properties of the digesta may lead to alterations on intestinal health parameters, especially the composition and diversity of the intestinal microbiota [15,31].

Based on the mechanisms of action and benefits of supplemental phytase and xylanase described above, it has been hypothesized that these enzymes could also play functional roles associated with the intestinal health of nursery pigs and broiler chickens. These functional roles could be related with a reduction in oxidative damage products and inflammatory cytokines, and a positive modulation of the intestinal microbiota [5,13,15,32]. This review focused on the characterization and discussion of the nutritional and functional roles of phytase and xylanase enhancing the intestinal health and growth of nursery pigs and broiler chickens.

## 2. Antinutritional Factors in Feeds for Nursery Pigs and Broiler Chickens

### 2.1. Phytic Acid

Phytate is a mixed salt of phytic acid (myo-inositol hexaphosphate; InsP6) present in plant-based feedstuffs and is constantly present in diets of monogastric animals [33,34]. Phytic acid is described as an antinutritional factor because it reduces the absorption and digestibility of trace minerals, such as Ca, zinc (Zn) and copper (Cu) forming insoluble and indigestible compounds [35,36,37,38]. Additionally, it can bind to amino acids, proteins, and enzymes like trypsin and α-amylase, inhibiting their activity and affecting protein and carbohydrate digestibility [39,40,41].

In general, around 70% of the P content in cereal grains and oil seeds used in diets for nursery pigs and broiler chickens is present in the form of phytic acid, which is not bioavailable for animal utilization [25,42]. Genetics, climate conditions, location, irrigation, soil nutrition, season, and fertilizer application may have an impact on phytic acid levels in the plant-based feedstuffs included in the diets [42]. The enzyme responsible for catalyzing the hydrolysis of phytic acid is phytase; however, monogastric animals lack on the production of endogenous phytase, which means that high levels of inorganic P sources were included in their diets to match their specific nutritional requirements [24,43]. As a result, there may be an increase in the costs associated with feed since inorganic P sources tend to be more expensive than other sources of P [44]. Additionally, environmental concerns are arising due to an increase in the excretion of undigested minerals in the manure, leading to soil and water contamination in the surrounding areas of pig and broiler chicken production systems [45,46].

During the digestion process for nursery pigs and broiler chickens, phytic acid is soluble under the acid pH of the gastric phase, which means that it is less susceptible to binding to other nutrients, reducing their bioavailability [24,47]. As the digestion process goes on, the digesta will move to the small intestine, where with different pH conditions, phytic acid will become insoluble increasing its ability to form indigestible phytate-complexes with other nutrients [34,48]. Thus, the main site for catalyzing the hydrolysis of phytic acid will be under acid pH in the stomach of pigs and proventriculus of broiler chickens, which will also provide better conditions for an optimal phytase activity [20,49,50].

### 2.2. Non-Starch Polysaccharides

The NSP have been described as antinutritional factors for nursery pigs and broiler chickens due to their inability to digest these compounds as a consequence of the absence of NSP degrading enzymes [5,12,51]. According to [12], around 30% of the composition of the main plant-based feedstuffs used in diets for nursery pigs and broiler chickens is NSP. The NSP content in plant-based feedstuffs can vary based on the plant genetics, environment, and storage conditions after harvesting [31]. Xylan, arabinoxylan, β-glucans, and cellulose are some of the main NSP present in those plant-based feedstuffs [52,53,54].

Non-starch polysaccharides are described as soluble and insoluble due to their structure, solubility, and impacts on the physicochemical properties of the digesta [55,56]. Cereal grains are described as containing higher levels of soluble NSP when compared to cereal by-products that contain higher levels of insoluble NSP [52,53]. Soluble NSP may alter the digesta viscosity, bulkiness, and passage rate due to its water holding capacity, which may lead to negative effects associated with nutrient digestibility and intestinal health [5,23,30,57]. Moreover, insoluble NSP may impact intestinal motility and transit time by acting as a barrier reducing the side activities of other endogenous enzymes and leading to detrimental effects on nutrient digestibility [29,56,58]. Plant-based feedstuffs used in diets for nursery pigs and broiler chickens can be classified as viscous and non-viscous based on the concentrations of soluble and insoluble NSP in their composition [52,54].

Xylan is described as the major class of hemicellulose and one of the main soluble NSP present in plant-based feedstuffs included in the diets of nursery pigs and broiler chickens [12,31,59]. It is also described as a family of structurally diverse NSP sharing a β-1,4-linked xylopyranose backbone as a common feature [60,61]. Classifications of xylan are usually based on the degree of substitution and type of side groups attached to the backbone [61,62]. Arabinoxylan (AX) is the most common form of xylan found in plant-based feedstuffs such as corn, sorghum, soybean meal, and wheat [63].

As previously mentioned, soluble NSP, especially xylan, can affect the digesta properties leading to negative impacts not only associated with nutrient digestibility but also on the intestinal health and mucosa-associated microbiota in the jejunum [1,64,65]. The negative effects of xylan on the digesta viscosity can be related to its chemical structure, molecular weight, swelling, and water-holding capacity [57,59]. When the digesta viscosity is increased, there are alterations to its passage rate and bulkiness [66,67]. An increase in the digesta bulk can cause a distention of the digestive tract walls leading to a greater secretion of satiety hormones and pancreatic secretions that can affect the daily intake of the animals [23,68].

In nursery pigs and broiler chickens, AX is poorly digested and can lead to a production of a viscous chyme in the small intestine resulting in an increase in the relative abundance of pathogenic bacteria, inflammatory and immune response, oxidative stress, and impairment of the intestinal barrier function [12,64,69]. An in vitro study simulating the digestion process of monogastric animals showed that the supplementation of xylanase, one of the NSP degrading enzymes, increased the availability of soluble minerals in corn, wheat, and soybean meal [70]. The authors attributed the positive effects on mineral availability to the inclusion of xylanase that reduced the antinutritional properties of xylan by breaking down the structural bonds of the xylan. The supplementation of xylanase may be a potential solution to the antinutritional properties of xylan and other NSP.

## 3. Phytase and Xylanase Enhancing the Intestinal Health of Nursery Pigs and Broiler Chickens

### 3.1. Phytase

#### 3.1.1. Characteristics and Mechanisms of Action

Phytases (myo-inositol hexakiphosphate phosphohydrolase) are a class of phosphatases enzymes responsible for catalyzing the hydrolysis and release of P of phytic acid present in plant-based feedstuffs used in diets for monogastric animals [24,50]. Throughout a series of stepwise dephosphorylation reactions, phytase will increase the bioavailability of P and will allow a reduction on the inclusion of inorganic P sources in the diets [71,72]. Phosphorus is an essential nutrient especially for pigs and broiler chickens playing important roles as a component of co-enzymes in important metabolic pathways, bone mineralization, and intestinal health [43,73,74]. Therefore, it is important to optimize the P utilization by the animals due to economic and environmental concerns associated with the inclusion of inorganic P sources in the diets [75]. The activity of each phytase is expressed as phytase units (FTU), where one FTU was defined as the amount of phytase needed for the release of 1 μmol of inorganic P per minute from an excess of 15 µM sodium phytate at pH 5.5 and 37 °C [76].

Phytase is a heat labile enzyme, which means that its optimal activity is more susceptible to be affected at high temperatures usually applied to ensure the quality of some feed mixing processes [24,77]. Some of the plant-based feedstuffs used in the diets for nursery pigs and broiler chickens expresses intrinsic phytase activity; however, in the case of corn and soybean meal, expresses a negligible activity [42,78]. A great portion of phytase activity in cereal grains is found at the aleurone layers; however, it can be reduced when exposed to heat during feed mixing [78].

At the same time that the gastric phase of digestion provides better conditions for the hydrolysis of phytic acid, it also provides better conditions for optimal phytase activity [24,49]. Generally, phytases tends to work under a pH range of 3 to 5.5, which is accomplished in the stomach of pigs and birds by acidic secretions [20,33]. Since the pH in the small intestine is more favorable for proteolytic enzymes, there is a possibility that phytase could be broken down or inactivated by other active endogenous proteases [24,49]. There are many other factors besides heat that can also affect the optimal activity of phytase, such as diet composition, storing conditions, and gastric pH and temperature [24,79].

With the improvement of manufacturing technologies in the last few years, there was an increase in the production and development of new types of phytase [20,44,80]. They can be classified based on the form (liquid, powder, granule), microorganism sources (bacterial, fungi, yeast) optimal pH range (acidic or alkaline), and the position of the carbon where the dephosphorylation of phytate will initiate (3- and 6-phytase products) [25,81,82]. Recently, phytase has been supplemented beyond the manufacturer standard dose levels, also known as “super dosing”, and showed potential results that are discussed as “extra-phosphoric effects” [83,84,85,86]. The “extra-phosphoric” effects due high doses of phytase are usually described as a greater release and uptake of P and other nutrients, and the subsequent generation of lower inositol esters and myo-inositol [33,38,83,86].

#### 3.1.2. 3- and 6-Phytase

Phytase can be classified based on the location of the carbon where the hydrolysis of the inositol ring will initiate [25,82,87]. The 3-phytase (EC 3.1.3.8) is described by initiating the dephosphorylation at the third carbon atom of the inositol ring, whereas the 6-phytase (EC 3.1.3.26) initiate at the sixth carbon atom, which may result in an enhanced hydrolysis of the inositol ring [88,89]. The pH changes towards a less acidic environment whereas the digesta goes to the small intestine can be a determinant factor for phytase activity because the pH in the small intestine is favorable for other endogenous proteolytic enzymes [24,49]. As a result, phytase may have natural decrease in its optimal activity and may be broken down by other endogenous proteases, becoming almost inactive in the small intestine [24,49].

Theoretically, 6-phytase may express optimal activity at a broader range of pH being more active and resistant than 3-phytase at the small intestine environment [19,24,50]. However, previous research has shown a variation among the results associated with growth performance, nutrient digestibility, and utilization between 3-phytase and 6-phytase, especially related to the growth stage when the animals were supplemented [84,90,91,92,93,94,95]. 

**Figure 1:**
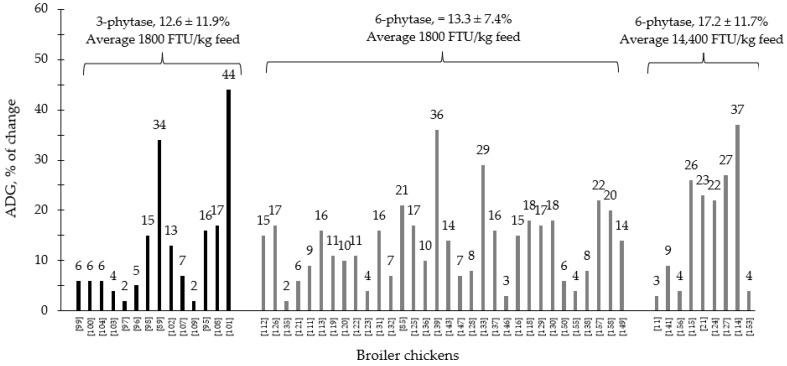
Changes in the average daily gain (ADG) of broiler chickens by 3- and 6-phytase supplementation among the studies displayed in Table 2 that showed changes in the ADG. The studies were selected from peer-reviewed literature available after 2000. The percentage of change refers to a statistically significant (*p* < 0.05) and tendency (0.05 ≤ *p* < 0.10) effects of phytase individually on the ADG reported from each respective study. The described effects were in comparison with treatments containing no phytase. The average changes in the ADG of broiler chickens by 3- and 6-phytase supplementation were calculated excluding the studies that showed no effect.

**Figure 2:**
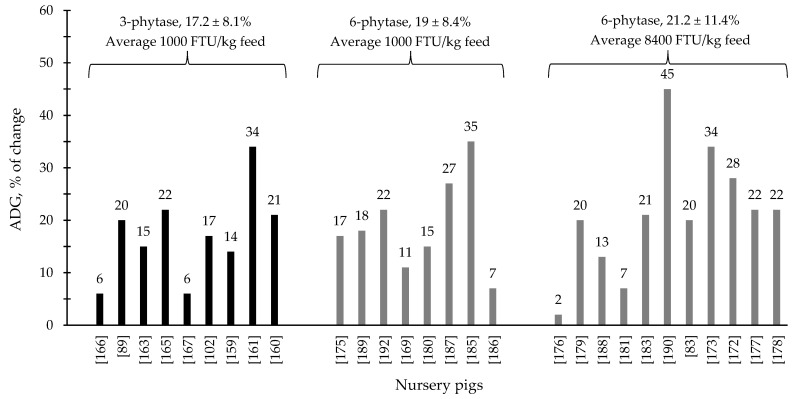
Changes in the average daily gain (ADG) of nursery pigs by 3- and 6-phytase supplementation among the studies displayed in Table 2 that showed changes in the ADG. The studies were selected from peer-reviewed literature available after 2000. The percentage of change refers to a statistically significant (*p* < 0.05) and tendency (0.05 ≤ *p* < 0.10) effects of phytase individually on the ADG reported from each respective study. The described effects were in comparison with treatments containing no phytase. The average changes in the ADG of nursery pigs by 3- and 6-phytase supplementation were calculated excluding the studies that showed no effect.

During the last few years, the supplementation of 3- and 6-phytase at different levels for swine and poultry at different stages of growth has shown consistent results, ranging from improvements on nutrient digestibility and growth performance to intestinal health parameters and modulation of the intestinal associated microbiota (Table 1 and Table 2). Generally, 6-phytase are more commonly used in swine and poultry production systems compared to 3-phytase, due to the benefits associated with the mode of action of this phytase. Based on the compiled data from Table 1 and Table 2, when 6-phytase was supplemented on average at 1800 and 14,400 FTU/kg feed showed respectively 13.3 and 17.2% of improvement in the ADG of broiler chickens compared to 12.6% from 3-phytase when supplemented 1800 FTU/kg feed (Figure 1). For nursery pigs, when 6-phytase was supplemented on average at 1000 and 8400 FTU/kg feed showed respectively 19 and 21.2% of improvement in the ADG compared to 17.2% from 3-phytase when supplemented 960 FTU/kg feed. (Figure 2). The changes in the ADG of broiler chickens and nursery pigs reported in Figure 1 and Figure 2 may indicate that 6-phytase could be supplemented at similar levels of 3- phytase and still provide greater changes in the ADG of the animals. Additionally, when 6-phytase was supplemented above the traditional levels it showed greater changes in the ADG of both broiler chickens and nursery pigs, when compared with the traditional supplemental levels (500–1000 FTU/kg feed).

#### 3.1.3. Supplementing Phytase beyond Traditional Levels

The concept of supplementing phytase beyond traditional levels, also known as “super dosing” phytase, emerged due to economic and environmental concerns associated with high amounts of inorganic P sources in diets of swine and poultry, as well as the quality and availability of the main plant-based feedstuffs, such as corn and soybean meal [50,85,194]. Thus, it was hypothesized that an inclusion of phytase 3-fold or greater than the recommended manufacturer dose could enhance the phytic acid dephosphorylation leading to an increase in the bioavailability of P and other nutrients, and the generation of myo-inositol and lower inositol phosphate esters [38,186,194].

Inositol can be found in mammalian cells and tissues in the forms of myo-inositol or phosphatidylinositol, and it is considered an important cellular mediator for signal transduction and a regulator of growth metabolism [195,196,197]. Additionally, it is known to have insulin-like effects, such as increasing the insulin sensitivity by enhancing the concentration of phosphatidylinositol (3,4,5)-trisphosphate (PIP3), and by increasing the insulin secretion by pancreatic β-cells [186,198,199]. Part of the benefits of the “extra phosphoric effects” are attributed by authors due to a greater generation of myo-inositol, and lower inositol phosphate esters more susceptible to hydrolysis and side activities of other endogenous enzymes [38,81,186,200]. The other part is attributed due to a greater generation and uptake of P, Ca, amino acids, and other nutrients that were trapped in the phytate-complexes and not bioavailable for the animals [38,185,186,194].

The “extra phosphoric effects” associated with supplementing phytase beyond traditional levels, are well documented and show a wide range of benefits, such as enhancement of nutrient digestibility, growth performance and intestinal health of pigs and broiler chickens [14,84,85,86,179,185,186,194,201,202].

#### 3.1.4. Effects of Supplementing Phytase on the Intestinal Health of Nursery Pigs and Broiler Chickens

**Figure 3:**
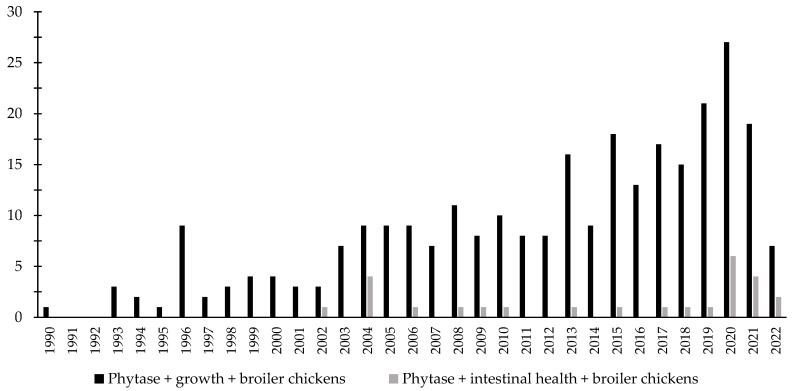
A list of the number of peer-reviewed papers found in the PubMed using different keywords. Black bars: phytase, growth, broiler chickens as key words; grey bars: phytase, intestinal health, broiler chickens as keywords.

**Figure 4:**
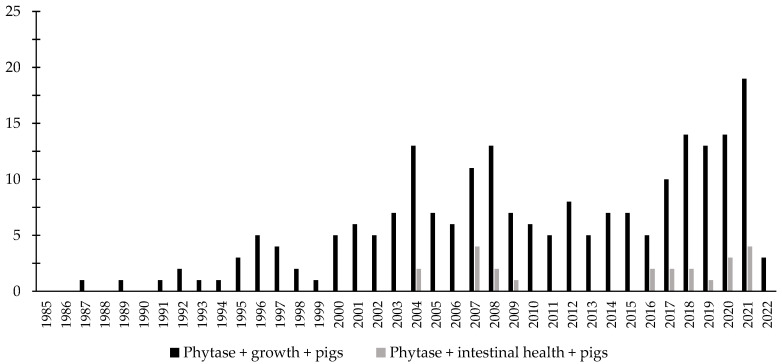
A list of the number of peer-reviewed papers found in the PubMed using different keywords. Black bars: phytase, growth, pigs as key words; grey bars: phytase, intestinal health, pigs as keywords.

Over the last few decades, there has been an increase in the interest in the investigation of the effects of phytase associated with the intestinal health of broiler chickens and nursery pigs (Figure 3 and Figure 4). Different studies were conducted aiming to investigate the effects of phytase on the intestinal health and microbiota of nursery pigs and broiler chickens [13,14,32,191,203,204,205,206]. The authors reported a wide range of positive effects of phytase supplementation not only related to growth performance, nutrient digestibility, and bone health, but especially related to intestinal health.

The authors of [14] reported a tendency for the relative abundance of Lactobacillus to increase and for *Helicobacter* and *Pelomonas* populations of the mucosa-associated microbiota to decrease in the jejunum of animals fed with phytase at 2000 FTU/kg feed. The authors attributed these effects to pH alterations possibly caused by a reduction in dietary Ca levels in the diets with phytase, and by the active hydrolysis of the phytate-complexes by phytase increasing the bioavailability of Ca and other minerals that can also affect the gastric and intestinal pH. Additionally, an increase was reported in the jejunal villus height, apparent ileal digestibility of nutrients, and bone health. Although the authors observed positive effects in the modulation of the mucosa-associated microbiota in the jejunum, no effects were observed in the inflammatory and oxidative stress parameters.

In another study conducted by [13], the authors observed increases in *Lactobacillus* and decreases in *Streptococcus* populations in the ileum of broiler chickens supplemented with phytase at 5000 FTU/kg feed. The authors explained these effects with alterations in the pH values in the crop, ileum, and caeca and short chain fatty acids production caused by the supplementation of phytase. Phytase supplementation increased the pH values in different gastric and intestinal sections, which may lead to a creation of a more acidic environment favoring beneficial bacteria and leading to a bacteriostatic effect against pathogenic bacteria. Additionally, an increase in the total short chain fatty acids, DL-lactate, and acetic acid in the ileum was reported with the addition of phytase, all of which are considered antioxidants and potential bacterial substrate. According to the authors, these findings coupled with the pH alterations in the crop, ileum, and caeca may be responsible for the positive effects observed in the microbiota of broiler chickens.

The authors of [207] reported increased concentrations of coenzyme Q10 in the liver of broiler chickens and turkeys when phytase was supplemented at 500 and 2500 FTU/kg feed. Coenzyme Q10 can enhance the antioxidant status of the animals by either targeting generated free radicals or through the regeneration of tocopherols and ascorbate, which are also antioxidant compounds [208,209]. Additionally, it can provide protective effects against metabolites originated from lipid peroxidation and protein oxidation [135,136], such as malondialdehyde (MDA) and protein carbonyl (PC). According to the authors, the increased bioavailability and uptake of nutrients, especially minerals, through the active hydrolysis of phytate by phytase may enhanced the antioxidant status of birds, by increasing the concentration of coenzyme Q10. On the other hand, the authors also believed that the greater bioavailability and uptake of metal ions from the minerals may have triggered the secretion of coenzyme Q10 as a response to increased oxidative damage.

In a study evaluating the effects of a corn-expressed phytase for nursery pigs [204], reported positive effects not only related to growth performance, but especially to intestinal health. Phytase supplementation increased villus height in the duodenum, jejunum, and on the villus height to crypt depth ratio in the duodenum. Additionally, it was also observed a tendency towards the reduction in the concentrations of tumor necrosis factor alpha (TNF-α) in the duodenum and MDA in the jejunum. These findings are in accordance with previously discussed studies reaffirming the positive effects of phytase through an active hydrolysis of phytate, and consequently a greater bioavailability and uptake of nutrients.

Another possible mechanism of phytase related to intestinal health, may be associated the “extra-phosphoric” effects aimed with the supplementation of high doses of this enzyme. As previously mentioned, these effects are characterized and discussed as a greater bioavailability and uptake of nutrients, and a greater generation of myo-inositol and lower inositol esters [83,186,194,200]. The authors of [202] observed an increase in plasma myo-inositol, and serum zinc and copper levels of nursery pigs when phytase was supplemented at 2500 FTU/kg feed. Although the authors did not measure other intestinal health related parameters, they observed positive effects on the growth performance of the pigs during their first ten days after weaning, which is one of the most critical periods for the pig. The authors suggested that the greater generation of myo-inositol and other essential minerals due to the higher doses of phytase, may positively affected the post-weaning performance of the animals.

In another study [83], also observed an increase in plasma myo-inositol and lower inositol esters concentrations, together with increased growth performance when phytase was supplemented above 500 FTU/kg feed for nursery pigs. An increase was also reported in the concentrations of glucose transporter type 4 (GLUT4) in the muscle plasma membranes and correlated with the increase in plasma myo-inositol concentrations. Glucose transporter type 4 is known to increase tissue glucose uptake during insulin signaling [210]. In this study, the authors hypothesized that phytase could be contributing to enhance the growth performance of nursery pigs through a different mechanism besides the increase in the bioavailability and uptake of nutrients, especially P.

Even though different studies reported positive effects on the intestinal health of nursery pigs and broiler chickens by supplementing phytase, there is still no consensus among authors regarding the mechanisms behind these benefits.

### 3.2. Xylanase

#### 3.2.1. Characteristics and Mechanisms of Action

Xylanases are described as a carbohydrase and classified under the glycosyl hydrolase enzyme family, which means that they catalyze the hydrolysis of glycosidic bonds in complex sugars compounds [12,211,212]. In this context, xylanase will catalyze the hydrolysis of 1,4-β-D-xylopyranosyl linkages of xylan, mitigating the antinutritional effects associated with this type of NSP [212,213]. As a result, xylanase can contribute to the reduction in digesta viscosity and release otherwise entrapped nutrients by facilitating increased access of other endogenous enzymes to their substrates leading to benefits in nutrient digestibility and growth performance [5,23,211,214]. Additionally, it can also contribute to the generation of fermented NSP-released compounds such as, xylooligosaccharides (XOS) and arabinoxylooligosaccharides (AXOS), that can increase the subsequent energy generation and play a prebiotic role that can lead to alterations in the composition and diversity of the intestinal microbiota [12,65,215].

Endo-xylanases (endo β-1,4-xylanase, EC 3.2.1.8) are described as hydrolyzing β-D-xylosidic bonds of the interior xylan backbone [212] and exo-xylanases (exo-β-1,4-xylanase, EC 3.2.1.37) by hydrolyzing the reducing and/or non-reducing end from long-chain xylo-oligomers [216]. From a commercial and industrial point of view, endo-xylanases are more commonly used in swine and poultry production due to its manufacturing process and availability [216]. Xylanase can be produced from different sources such as, yeast, fungi, and bacteria, which can determine the standard supplemental level, optimal conditions, and enzyme activity [212]. The genetic and functional information, as well as the classification of different xylanases under the glycoside hydrolase family, can be found at CAZy database (http://www.cazy.org, accessed on 16 November 2022). The activity of xylanase can be expressed as xylanase unit (XU), where one unit will liberate 1 µmol of reducing sugar measured as xylose equivalents from xylan per minute under standard assay conditions.

Besides optimal temperature and pH conditions, it is important to ensure the presence of the targeted substrate in the diets in order to optimize dietary enzyme activity and enhance its associated benefits. Monogastric animals, especially swine and poultry, do not endogenously secrete the necessary enzymes to hydrolyze xylan [12], which according to [65] may require nine enzymes to completely hydrolyze and saccharify it. The main plant-based dietary feedstuffs (corn, soybean meal, wheat, sorghum) can contain up to 30% of NSP in their composition. Increased price and availability concerns coupled with relatively high levels of NSP among these feedstuffs offers opportunities for the supplementation of xylanase in swine and poultry diets to aid in the mediation of deleterious effects induced by NSP due to the inability of the animal to produce endogenous enzymes to properly hydrolyze xylan. 

Compared to growing and finishing pigs, which also shows potential results supplementing xylanase, nursery pigs have an immature digestive system and capacity as a result of the wide range of stressors affecting them after weaning. As the pigs grow during nursery phase, the levels of NSP in their diets tend to increase since there will be a greater inclusion of plant-based feedstuffs. Recently, more studies are being conducted evaluating the supplementation xylanase for nursery pigs, and they have been showing potential results, especially related to enhancements in nutrient digestibility and intestinal health [5,23,29,30]. These findings may raise an opportunity for increasing the inclusion xylanase in diets for nursery pigs.

**Figure 5:**
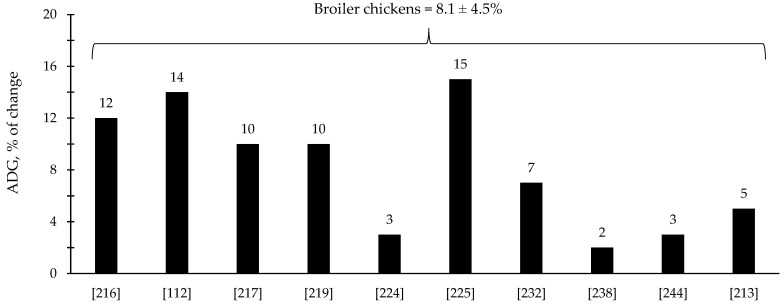
Changes in the average daily gain (ADG) of broiler chickens by xylanase supplementation among the studies displayed in Table 3 that showed changes in the ADG. The studies were selected from peer-reviewed literature available after 2000. The percentage of change refers to a statistically significant (*p* < 0.05) and tendency (0.05 ≤ *p* < 0.10) effects of xylanase individually on the ADG reported from each respective study. The described effects were in comparison with treatments containing no xylanase. The average changes in the ADG of broiler chickens by xylanase supplementation were calculated excluding the studies that showed no effect.

**Figure 6:**
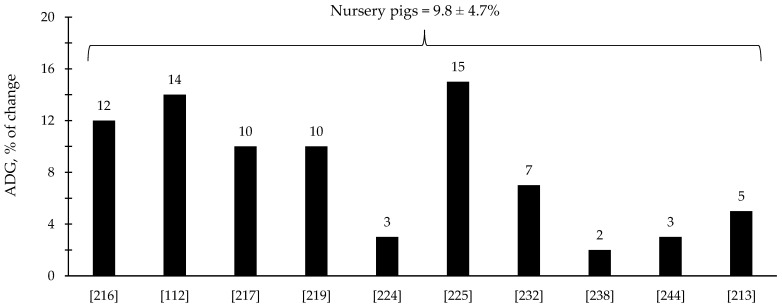
Changes in the average daily gain (ADG) of nursery pigs by xylanase supplementation among the studies displayed in Table 3 that showed changes in the ADG. The studies were selected from peer-reviewed literature available after 2000. The percentage of change refers to a statistically significant (*p* < 0.05) and tendency (0.05 ≤ *p* < 0.10) effects of xylanase individually on the ADG reported from each respective study. The described effects were in comparison with treatments containing no xylanase. The average changes in the ADG of nursery pigs by xylanase supplementation were calculated excluding the studies that showed no effect.

The efficacy of xylanase supplementation in swine and poultry diets has been relatively variable in comparison with phytase with some studies reporting improvements in the reduction in digesta viscosity and growth performance as well as positive alterations in intestinal health and microbiota; however, other studies reported no effects (Table 3 and Table 4). These inconsistent results from xylanase supplementation could be due to a myriad of factors such as animal species and growth stage, diet composition, supplementation level, and xylanase properties. Based on the compiled data from Table 3 and Table 4, xylanase supplementation showed on average 8.1% improvement in the ADG of broiler chickens (Figure 5) and 9.8% improvement in the ADG of nursery pigs (Figure 6).

**Table 3:** A list of studies describing effects of xylanase supplemented individually at different levels in diets for broiler chickens.

Duration, Day of Age	Activity	% Change *	Reference **
1–42	ND ^1^	Final body weight (2%), ADFI ^2^ (−2%), tissue protein content (14%), gizzard weight (−8%), duodenum-jejunum weight (−8%), ileal digesta viscosity (ND), ileum lactic acid bacteria (4%)	[217]
1–25	0–200 FXU/kg feed	ADG ^3^ (12%), FCR ^4^ (−9%), excreta moisture (−5%), jejunum arabinose (80%), jejunum xylose (95%), ileum arabinose (88%), ileum xylose (97%), duodenum digesta viscosity (−33%), jejunum digesta viscosity (−49%)	[218]
1–22	0–1000 XU/kg feed	ADG (14%), ADFI (10%), FCR (−5%), duodenum digesta viscosity (−29%), jejunum digesta viscosity (−23%), ileum digesta viscosity (−39%), jejunum weight (−16%), jejunum length (−16%), jejunum crypt depth (−13%)	[113]
1–41	0–2000 U/kg feed	ADG (10%), FCR (−6%), in-vitro intrinsic viscosity (−38%)	[219]
1–28	0–500 U/kg feed	Jejunum digesta viscosity (−85%)	[220]
1–18	ND	ADG (10%), ADFI (16%), ileum length (−30%), jejunum crypt depth (−19%)	[221]
1–21	0–1000 XU/kg feed	FCR (−2%), AME ^5^ (3%)	[222]
7–28	0–500 U/kg feed	G:F ^6^ (8–16%), jejunum digesta viscosity (−18%), DM ^7^ digestibility (27–37%)	[223]
1–28	0–2500 GXU/kg feed	ADFI (9%), jejunum digesta viscosity (−11%), cecum arabinose concentration (59%)	[224]
1–43	0–16,000 U/kg feed	FCR (−3%), N ^8^ digestibility (3%), ileal digestible energy digestibility (3%), threonine digestibility (5%), lysine digestibility (3%)	[225]
1–24	0–200 FXU/kg feed	ADG (3%), G:F (4%), jejunum digesta viscosity (−21%), DM retention (10%), CP ^9^ retention (13%), energy retention (9%)	[226]
1–42	0–2000 U/kg feed	ADG (15%), FCR (−11%), cecum *Salmonella* prevalence (−62%)	[227]
1–35	ND	Feed conversion ratio (−3%), starch digestibility (4%), fat digestibility (3%), AME retention (4%)	[228]
1–43	0–16,000 BXU/kg feed	FCR (−3%)	[229]
1–43	0–16,000 XU/kg feed	Serum insulin (%), serum peptide YY (61%), cecal VFA ^10^ (−4%)	[230]
1–35	0–250 FXU/kg feed	Ileum viscosity (−20%), ileum xylose concentration (−14%), jejunum protein content (28%)	[231]
1–32	0–32,000 BXU/kg feed	No effects on the evaluated parameters	[232]
1–50	0–16,000 BXU/kg feed	FCR (−7%), DM digestibility (9%), ileal digestible energy (7%), N digestibility (4%), cecum temperature (4%)	[233]
1–43	0–1250 XU/kg feed	ADG (7%), FCR (−5%), jejunum viscosity (−49%), cecum acetic acid concentration (35%), fat digestibility (5%), CP digestibility (4%), DM retention 3%), fat retention (6%), P ^11^ retention (6%), NDF ^12^ retention (14%), ADF ^13^ retention (42%), AME (2%)	[234]
1–21	0–5500 U/kg feed	FCR (−7%)	[235]
1–49	0–32,000 BXU/kg feed	FCR (−6%), energy digestibility (6%), gizzard weight (16%), cecum propionic acid concentration (22%), cecum caproic acid concentration (24%)	[236]
7–21	0–2000 U/kg feed	AME (1%), DM retention (2%), fat digestibility (2%)	[237]
1–42	0–160,000 BXU/kg feed	Gizzard weight (17%), gizzard length (19%)	[238]
1–42	0–2000 XU/kg feed	FCR (−2%), ileal digestible energy (8%), starch digestibility (1%), N digestibility (5%), GE digestibility (8%), ileum total NSP ^14^ concentration (−26%)	[239]
1–36	0–5625 XU/kg feed	ADG (2%), GE ^15^ digestibility (3%), CP digestibility (4%), DM digestibility (1%), RA ^16^ of ileal *Lactobacillus* (12%), cecal *Lactobacillus* (11%), ileal *Escherichia coli* (−15%), and cecal *Escherichia coli* (−11%), duodenum villus height (8%), jejunum villus height (10%), ileum villus height (12%)	[240]
1–42	0–16,000 BXU/kg feed	FCR (−4%)	[241]
1–41	0–24,000 BXU/kg feed	Mortality (−54%)	[242]
1–42	0–16,000 BXU/kg feed	FCR (−3%), duodenum acetic acid concentration (12%)	[243]
1–42	0–200 FXU/kg feed	FCR (−1%), jejunum digesta viscosity (−40%), ileum digesta viscosity (−58%)	[244]
1–29	0–160,000 BXU/kg feed	FCR (−8%), copper digestibility (44%)	[245]
1–30	0–32,000 BXU/kg feed	Ileal frutose (31%), ileal arabinose (29%), ileal galactose (33%)	[215]
1–33	0–11,250 XU/kg feed	ADG (3%), FCR (−4%), DM digestibility (4%), GE digestibility (4%), ileum digesta viscosity (−12%), duodenum villus height (8%), jejunum villus height (9%)	[246]
1–43	0–16,000 BXU/kg feed	ADG (5%), final body weight (6%), acetate (27%), total SCFA ^17^ (24%)	[214]

* The described effects were in comparison with treatments without xylanase. Described effects were considered significant with *p* < 0.05 and tendency with 0.05 ≤ *p* < 0.10. ** The references were selected from peer-reviewed literature available after 2000. ^1^ No data. ^2^ Average daily feed intake. ^3^ Average daily gain. ^4^ Feed conversion ratio. ^5^ Apparent metabolizable energy. ^6^ Gain to feed ratio. ^7^ Dry matter. ^8^ Nitrogen. ^9^ Crude protein. ^10^ Volatile fatty acids. ^11^ Phosphorus. ^12^ Neutral detergent fiber. ^13^ Acid detergent fiber. ^14^ Non-starch polysaccharides. ^15^ Gross energy. ^16^ Relative abundance. ^17^ Short chain fatty acids.

**Table 4:** A list of studies describing effects of xylanase supplemented individually at different levels in diets for nursery pigs.

Duration, Day of Age	Activity	% Change *	Reference **
35–70	0–4000 XU/kg feed	DM ^1^ fecal digestibility (1%), N ^2^ digestibility (2%)	[247]
7–28	0–5600 EXU/kg feed	No effects on the evaluated parameters	[248]
21–56	ND ^3^	FCR ^4^ (−3%), CP ^5^ digestibility (4%), fat digestibility (2%), starch digestibility (1%), total amino acids digestibility (3%), leucine digestibility (2%), isoleucine digestibility (4%), jejunum digesta viscosity (−76%), colon digesta viscosity (−81%), jejunum acetate concentration (27%), jejunum propionate concentration (30%), total conjugated/deconjugated bile acids (33%)	[249]
ND	0–1500 U/kg feed	CP digestibility (2%), crude ash digestibility (2%), Ca ^6^ digestibility (3%), P ^7^ digestibility (1%), ADF ^8^ digestibility (9%)	[250]
ND^1^	0–1400 LXU/kg feed	NDF ^9^ digestibility (36%), DM digestibility (17%), GE ^10^ digestibility (15%), OM ^11^ digestibility (15%), jejunum digesta viscosity (−14%)	[29]
ND	0–0.01%	ADG ^12^ (5%), G:F ^13^ (4%), DM digestibility (3%), N digestibility (4%), GE digestibility (3%), fecal *Lactobacillus* (1%)	[69]
28–56	0–4000 XU/kg feed	DM digestibility (2%), NDF digestibility (23%), P digestibility (29%)	[251]
21–49	0–4000 XU/kg feed	ADG (14%), DM digestibility (4%), CP digestibility (7%), NDF digestibility (19%), ADF digestibility (15%), Ca digestibility (22%), P digestibility (14%), fecal *Lachnospiraceae* (−50%)	[252]
23–43	0–1500 EPU/kg feed	SCFA ^14^ (20%), acetate (32%), propionate (19%), total NSP ^15^ digestibility coefficient (15%), arabinoxylan digestibility coefficient (38%), GE digestibility coefficient (8%), duodenum villus height (11%), jejunum crypt proliferation rate (17%), jejunum claudin (57%), jejunum occludin (75%), jejunum zonula occludens 1 (80%), jejunum digesta viscosity (−26%)	[30]
21–45	0–45,000 XU/kg feed	ADG (6%), jejunal digesta viscosity (−13%), jejunum mucosal MDA ^16^ (−17%), jejunum crypt depth (−10%), jejunum crypt cell proliferation (−15%)	[23]
18–53	0–16,000 BXU/kg feed	Ileum pH (6%), colon pH (3%), CP digestibility (7%), DM digestibility (3%), Ca digestibility (16%), P digestibility (8%), colon propionic acid concentration (10%)	[253]
21–41	0–1500 EPU/kg feed	ADG (7%), jejunal digesta viscosity (−14%), plasma TNF-α ^17^ (−36%), GE digestibility (6%), NDF digestibility (22%), duodenum crypt depth (11%)	[58]
23–65	0–16,000 BXU/kg feed	Ileum *Clostridiaceae* (−10%), cecum *Lactobacillaceae* count (36%)	[254]
21–63	0–16,000 XU/kg feed	ADG (9%), DM digestibility (4%), GE digestibility (3%), N digestibility (3%) and P digestibility (15%), RA ^18^ fecal *Veillonella* spp. (−33%)	[255]
21–59	0–1760 XU/kg feed	Jejunal digesta viscosity (−23%), jejunum mucosal MDA (−39%), jejunum mucosal PC ^19^ (−15%), jejunum villus height (13%), NDF digestibility (6%), EE ^20^ digestibility (6%), RA of jejunum *Cupriavidus* (−43%), *Megasphaera* (−82%)*, Succinivibrio* (73%), and *Pseudomonas* (63%)	[5]
28–63	0–135,000 U/kg feed	ADG (18%), ADFI (−2%), G:F (20%), diarrhea rate (−61%), DM digestibility (4%), CP digestibility (6%), EE digestibility (8%), NDF digestibility (2%), ADF digestibility (7%), GE digestibility (30%), starch digestibility (27%), N digestibility (26%), jejunum villus height (15%), feces propionate concentration (41%), fecal butyrate concentration (35%), feces ammonia concentration −26%)	[256]

* The described effects were in comparison with treatments without xylanase. Described effects were considered significant with *p* < 0.05 and tendency with 0.05 ≤ *p* < 0.10. ** The references were selected from peer-reviewed literature available after 2000. ^1^ Dry matter. ^2^ Nitrogen. ^3^ No data. ^4^ Feed conversion ratio. ^5^ Crude protein. ^6^ Calcium. ^7^ Phosphorus. ^8^ Acid detergent fiber. ^9^ Neutral detergent fiber. ^10^ Gross energy. ^11^ Organic matter. ^12^ Average daily gain. ^13^ Gain to feed ratio. ^14^ Short chain fatty acids. ^15^ Non-starch polysaccharides. ^16^ Malondealdehyde. ^17^ Tumor necrosis factor alpha. ^18^ Relative abundance. ^19^ Protein carbonyl. ^20^ Ether extract.

#### 3.2.2. Effects of Supplementing Xylanase on the Intestinal Health of Nursery Pigs and Broiler Chickens

**Figure 7:**
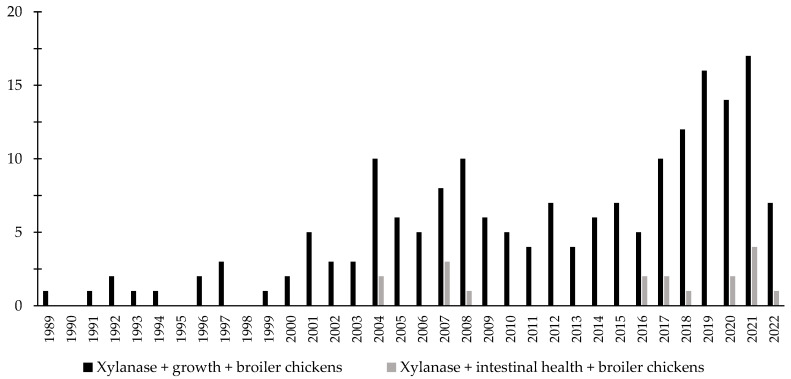
A list of the number of peer-reviewed papers found in the PubMed using different keywords. Black bars: xylanase, growth, broiler chickens as key words; grey bars: xylanase, intestinal health, broiler chickens as keywords.

**Figure 8:**
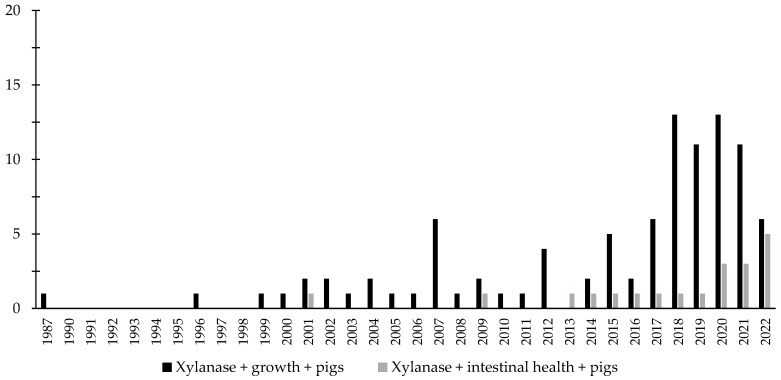
A list of the number of peer-reviewed papers found in the PubMed using different keywords. Black bars: xylanase, growth, pigs as key words; grey bars: xylanase, intestinal health, pigs as keywords.

Throughout the last few years, there has been an increase in the interest in the investigation of the effects of xylanase related with the intestinal health of broiler chickens and nursery pigs (Figure 7 and Figure 8). Several studies were conducted investigating the effects of xylanase on the intestinal health and microbiota of nursery and broiler chickens [5,15,213,214,215,242]. The authors reported a wide range of positive effects of xylanase supplementation not only related to growth performance and nutrient digestibility, but especially related to intestinal health.

**Figure 9:**
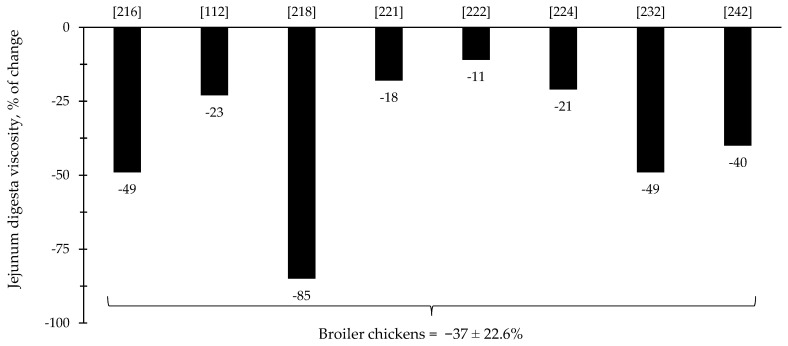
Changes in the jejunum digesta viscosity of broiler chickens by xylanase supplementation among the studies displayed in Table 3 that showed changes in the jejunal digesta viscosity. The studies were selected from peer-reviewed literature available after 2000. The percentage of change refers to a statistically significant (*p* < 0.05) and tendency (0.05 ≤ *p* < 0.10) effects of xylanase on the jejunal digesta viscosity reported from each respective study. The described effects were in comparison with treatments containing no xylanase. The average changes in the jejunal digesta viscosity of broiler chickens by xylanase supplementation were calculated excluding the studies that showed no effect.

**Figure 10:**
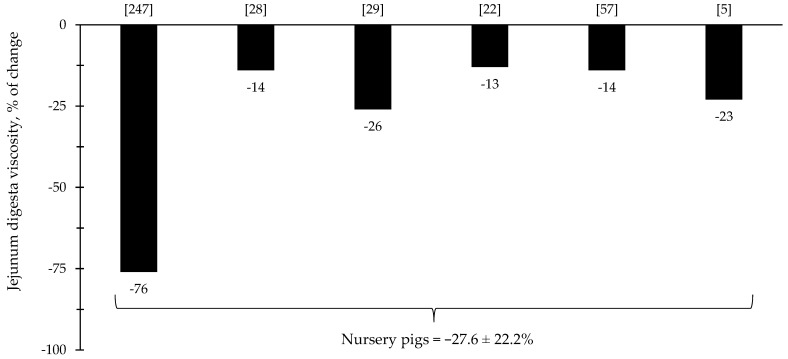
Changes in the jejunum digesta viscosity of nursery pigs by xylanase supplementation among the studies displayed in Table 4 that showed changes in the jejunal digesta viscosity. The studies were selected from peer-reviewed literature available after 2000. The percentage of change refers to a statistically significant (*p* < 0.05) and tendency (0.05 ≤ *p* < 0.10) effects of xylanase on the jejunal digesta viscosity reported from each respective study. The described effects were in comparison with treatments containing no xylanase. The average changes in the jejunal digesta viscosity of nursery pigs by xylanase supplementation were calculated excluding the studies that showed no effect.

The reduction in digesta viscosity and increase in nutrient digestibility are described as some of the primarily established benefits of xylanase supplementation for pigs and broiler chickens. Most of the studies for broiler chickens showed consistent improvements related to the reduction in jejunal digesta viscosity (=37%), which may account for other benefits reported in each of the respective described studies (Figure 9). For nursery pigs, xylanase also showed potential improvements related to the reduction in jejunal digesta viscosity (27.6%; Figure 10). These benefits may contribute for the expression of other possible benefits, such as, reduction in the oxidative stress, positive modulation of the intestinal microbiota, and enhancement of growth performance. However, there were studies that even reporting a reduction in the digesta viscosity did not report improvements on the growth performance [5,29].

The authors of [5] reported reduced digesta viscosity and increased apparent ileal digestibility (AID) of neutral detergent fiber (NDF), crude protein (CP), and ether extract (EE) of nursery pigs supplemented with increasing levels of xylanase (0, 220, 440, 880, 1760 XU/kg feed), whereas no effects on the overall growth performance. Firstly, the authors attributed these benefits to the depolymerization of the β-1,4 xylan bonds into shorter chain that possibly led to alterations in the physicochemical properties of the digesta and consequently reducing its viscosity and bulkiness. A higher digesta viscosity can alter the passage rate and reduce the interaction between digesta and other endogenous enzymes and lipid emulsifiers. Secondly, they also believed that the depolymerization of the xylan bonds led to an increase in the release trapped nutrients and fermentable NSP-released compounds. The results from [5] agree with other studies that also reported supplemental effects of xylanase reducing digesta viscosity and increasing digestibility of both nursery pigs and broiler chickens [23,58,240]. A reduction in digesta viscosity and bulkiness, and subsequent generation of trapped nutrients and fermentable NSP-released compounds bioavailability may contribute to the positive results observed in growth performance and nutrient digestibility, but especially on the modulation of mucosa-associated microbiota in the jejunum and jejunal oxidative stress and morphology parameters.

Moreover, a reduction was reported in the MDA and PC concentrations in the jejunal mucosa as indicators of reduced oxidative stress. These results are in agreement with [23] and [213], whereas [30] and [58] did not observe any xylanase-related effects to oxidative stress status. Overall, there is still no consensus about the specific mechanisms of xylanase modulating the oxidative stress and enhancing antioxidant capacity. The authors of [140] suggested that a potential mechanism could be due to an increasing fragmentation of the xylan structure and subsequent generation of fermentable NSP-released compounds, more specifically comprised of phenolic compounds such as, ferulic acid. Ferulic acid can play antioxidant [257] and antimicrobial functions [258] and was shown to be correlated with oxidative stress status of pigs [259] and with a reduction in pathogenic bacteria concentration in the feces [260].

The potential mechanism of xylanase associated with the reduction in inflammatory response parameters remains unclear. The authors of [58] reported decreased plasma concentration of TNF-α and peptide YY (PYY) of nursery pigs. The authors believed that the active hydrolysis of the β-1,4 xylan bonds generating oligosaccharides coupled with the observed reduction in digesta viscosity may contributed to the reduction in pro-inflammatory cytokines such as TNF-α, that are known as mediators of the immune response. The reduction in the plasma concentration of PYY by xylanase in nursery pigs by [261]; however, it did not show the same effects for broiler chickens [230]. The supplementation of xylanase has shown consistent results regarding the reduction digesta viscosity and increasing nutrient digestibility between nursery pigs and broiler chickens [5,23,70,215,219]. On the other hand, nursery pigs and broiler chickens may express different responses to the use of xylanase on the intestinal health parameters.

According to [4], the intestinal microbiota can be altered at different taxonomic levels based on the given fermentable substrate. In this context, the short chain fatty acids, XOS, and AXOS potentially released from the xylan structure would be rapidly fermented by beneficial bacteria populations such as, lactic acid bacterial, and may exert prebiotic functions [13]. The results from [5] showed a tendency on the reduction in the relative abundance (RA) of *Cupriavidus* and *Megasphera* and on the increase in *Succinivibrio* and Pseudomonas in jejunal mucosa of nursery pigs. The authors of [262] found a correlation between a low abundance of *Succinivibrio* with gastrointestinal disorders and loss of intestinal integrity in the colon of humans, and [263] reported this genus degrading cellulose and hemicellulose. These shifts in the jejunal bacterium population may indicate changes in the intestinal environment and substrates towards a healthier state, as a consequence of the positive effects observed in the nutrients digestibility and reduced viscosity. The authors of [15] also observed positive effects of xylanase supplementation in the modulation of the microbiota by increasing the RA of *Lactobacillus* and *Bifidobacterium*, and decreasing *Streptococcus* and *Turicibacter* in the ileum digesta; and increasing *Bifidobacterium* and decreasing *Escherichia-shigella* in the ileum mucosa of growing pigs. Regarding the effects of xylanase on the intestinal microbiota of broiler chickens [240], observed a decrease in the RA of *Escherichia coli* and an increase in *Lacbotobacillus* in the cecum, whilst [214] reported an increase in *Rumonococcaceae* also in the cecum.

Although the effects of xylanase positively modulating the intestinal microbiota of nursery pigs and broiler chickens show great potential, further investigation is still desired in order to elucidate the specific mechanisms of action. A positive modulation of the intestinal microbiota even at different levels and sections can contribute to enhance positive effects associated with other parameters and overall biological response. An increase in the RA of selected beneficial bacteria may increase the endogenous secretion of other degrading enzymes and short chain fatty acids, collectively enhancing nutrient digestibility, intestinal health, and growth performance of nursery pigs and broiler chickens. Even showing variable results, the supplementation of xylanase also shows positive results associated with the intestinal health of nursery pigs and broiler chickens. However, there is still no consensus among authors regarding the different mechanisms of action of xylanase among the evaluated parameters, animal species, and inclusion levels.

## 4. Conclusions

In conclusion, 6-phytase seems to be more effective and to provide a wider range of benefits compared to 3-phytase, especially related to growth performance. The supplementation of 6-phytase for nursery pigs and broiler chickens reaffirmed the positive results related to enhancement of nutrient digestibility, bone health, and growth performance, but also showed functional roles reducing oxidative stress parameters, and positively modulating the intestinal microbiota. Supplementing phytase beyond traditional levels (500 to 1000 FTU/kg feed), so-called “super-dosing” (above 1000 FTU/kg feed), showed a more active hydrolysis of phytic acid providing “extra-phosphoric effects” that were further reflected in improvements in intestinal and bone health, nutrient digestibility, growth performance of nursery pigs and broiler chickens.

The supplementation of xylanase can effectively reduce the digesta viscosity of nursery pigs and broiler chickens, which in turn could lead to potential benefits associated with the reduction in immune and oxidative stress parameters, positive modulation of intestinal microbiota, and enhancement of nutrient digestibility. Therefore, the growth performance of the animals can be potentially enhanced by supplementing xylanase.

Overall, the supplementation of phytase and xylanase in diets for nursery pigs and broiler chickens reaffirmed their nutritional roles enhancing nutrient digestibility and growth performance, whilst also playing functional roles reducing the oxidative stress response, and possibly through the modulation of the mucosal microbiota in the small intestine. As a result, it could contribute to a reduction in the feed costs by allowing the use of a wider range of feedstuffs without compromising the optimal performance of the animals, as well as the environmental concerns associated with a poor hydrolysis of antinutritional factors present in the diets for nursery pigs and broilers chickens.

## Figures and Tables

**Table 1:** A list of studies since 2000 describing effects of 3- and 6-phytase supplemented individually at different levels in diets for broiler chickens.

Duration, Day of Age	Type	Activity, FTU/kg Feed	% Change *	Reference **
14–35	3-phytase	0–2500	ADG ^1^ (16%), ADFI^2^ (7%), Ca ^3^ digestibility (12%), P ^4^ digestibility (16%)	[96]
7–28	3-phytase	0–1000	ADG (5%), G:F ^5^ (4%), lysine digestibility (6%), arginine digestibility (5%), AME ^6^ (3%)	[97]
21–40	3-phytase	0–800	ADG (2%), FCR ^7^ (−3%), bone ash (4%)	[98]
1–43	ND ^8^	0–1000	ADG (15%), ADFI (8%), FCR (−7%), bone ash (15%), bone Ca (8%), plasma P (45%)	[99]
1–43	3-phytase	0–500	ADG (6%), Ca retention (13%), P retention (9%), bone ash (5%), plasma Ca (−2%), plasma P (7%)	[100]
8–22	3-phytase	0–1000	ADG (34%), G:F (16%), bone ash (14%)	[90]
1–19	3-phytase	0–600	ADG (6%), ADFI (3%), Ca retention (8%), P retention (11%), tibia ash (2%), bone Ca (3%), bone P (2%), plasma Ca (5%), plasma P (5%)	[101]
1–17	3-phytase	0–12,000	ADG (44%), ADFI (36%), plasma P (65%), bone ash (36%), Ca retention (15%), P retention (36%), phytate P disappearance (57%), AME (6%)	[102]
1–42	3-phytase	0–1000	ADG (13%), ADFI (9%), G:F (5%), bone ash (9%), DM ^9^ digestibility (2%), energy digestibility (2%), P digestibility (14%)	[103]
1–42	3-phytase	0–750	ADG (4%), ADFI (3%), bone ash (3%)	[104]
1–7	3-phytase	0–600	ADG (6%), ADFI (6%), bone ash (5%)	[105]
1–51	3-phytase	0–600	ADG (−3%), ADFI (−3%), G:F (−1%)	[106]
22–38	3-phytase	0–500	Plasma P (7%), P retention, Ca retention (11%), bone ash (1%)	[107]
22–42	3-phytase	0–1000	ADG (7%), G:F (8%), plasma P (48%), bone ash (11%)	[108]
22–42	3-phytase	0–4000	ADG (17%), ADFI (7%), FCR (−10%), plasma Ca (−16%)	[109]
1–35	3-phytase	0–1000	ADG (2%), ADFI (2%), FCR (−4%), bone Ca (31%), bone P (28%), liver weight (33%), heart weight (−25%)	[110]
7–25	6-phytase	0–800	AME (4%), P digestibility (28%), N ^10^ digestibility (3%), lysine digestibility (2%), threonine digestibility (5%), DM retention (3%), P retention (10%), N retention (6%)	[111]
1–43	6-phytase	0–1000	ADG (9%), ADFI (8%), tibia ash (7%), toe ash (8%)	[112]
1–22	6-phytase	0–500	ADG (15%), ADFI (12%), FCR (−3%), jejunum digesta viscosity (−17%), ileum digesta viscosity (−25%), duodenum villus height (8%), jejunum crypt depth (−11%), jejunum goblet cell number (−37%)	[113]
1–22	6-phytase	0–1000	ADG (16%), ADFI (11%), tibia ash (6%), Ca digestibility (12%), P digestibility (13%)	[114]
1–15	6-phytase	0–24,000	ADG (37%), ADFI (13%), FCR (−28%), AME (5%), bone ash (20%), P retention (22%), phytate P digestibility (28%), lysine digestibility (2%), threonine digestibility (2%), total indispensable amino acid digestibility (4%)	[115]
9–23	6-phytase	0–10,000	ADG (26%), bone ash (21%)	[116]
1–22	6-phytase	0–2500	ADG (15%), ADFI (16%), AME (2%)	[117]
1–17	6-phytase	0–5000	FCR (−4%), tibia ash (5%), gizzard proximal pH (11%), jejunum pH (2%), ileum proximal pH (1%), ileum distal pH (6%), lysine digestibility (2%), arginine digestibility (2%), valine digestibility (4%)	[118]
1–29	6-phytase	0–2500	ADG (18%), ADFI (15%), G:F (4%), AME (1%), sialic acid concentration (−8%), threonine “metabolisability” (2%)	[119]
1–29	6-phytase	0–1000	ADG (11%), ADFI (5%), FCR (−7%), erythrocyte-antibody complement cells (15%), CD4^+^ T lymphocyte subset (13%), CD8^+^ T lymphocyte subset (12%), Newcastle disease antibody (33%)	[120]
7–21	6-phytase	0–12,500	ADG (23%), FCR (−15%), free retinol concentration (37%), liver coenzyme Q10 (39%)	[22]
1–22	6-phytase	0–1000	ADG (10%), ADFI (5%), G:F (6%), proventriculus pepsin activity (11%), jejunum alanyl aminopeptidase activity (9%), duodenum methionyl-aminopeptidase gene expression (5%), duodenum ghrelin gene expression (10%)	[121]
1–22	6-phytase	0–600	ADG (6%), bone ash (10%), P digestibility (31%), phytate P digestibility (29%), AME (4%), DM retention (4%), GE ^11^ retention (4%), N retention (7%), P retention (22%)	[122]
1–22	6-phytase	0–1000	ADG (11%), DM digestibility (9%), CP ^12^ digestibility (7%), P digestibility (41%)	[123]
1–22	6-phytase	0–1000	ADG (4%), DM digestibility (17%), N digestibility (9%), DM retention (9%), N retention (10%), Ca retention (23%)	[124]
7–22	6-phytase	0–12,500	ADG (22%), G:F (16%), liver carotenoids (39%)	[125]
8–23	6-phytase	0–1500	ADG (17%), protein gain (16%)	[126]
1–22	6-phytase	0–500	ADG (17%), ADFI (17%), mortality (−62%), BBS ^13^ (40%), bone ash (9%), Ca utilization (7%), P digestibility (20%), P utilization (11%)	[127]
7–18	6-phytase	0–12,500	ADG (27%), DM ADFI (19%), G:F (10%), AME (2%), DM digestibility (5%), carcass weight (22%), retained carcass protein (29%), retained carcass fat (36%), lysine digestibility (7%), threonine digestibility (11%)	[128]
1–22	6-phytase	0–2000	ADG (8%), ADFI (7%), bone ash (11%), bone mineral content (20%), bone mineral density (13%), P absorption (12%), P retention (16%), Ca retention (14%), lysine digestibility (2%), threonine digestibility (10%)	[129]
1–22	6-phytase	0–2500	ADG (17%), ADFI (17%), tibia ash (9%), bone Ca (16%), bone P (12%), Ca digestibility (45%), P digestibility (4%), N digestibility (5%), energy digestibility (4%)	[130]
1–22	6-phytase	0–2500	ADG (18%), ADFI (13%), G:F (6%), AME intake (13%)	[131]
15–57	6-phytase	0–1000	ADG (16%), ADFI (10%), and G:F (6%), tibia ash (18%), tibia weight (33%), Ca digestibility (15%) and P digestibility (23%)	[132]
1–22	6-phytase	0–1000	ADG (7%), Ca digestibility (21%), P digestibility (19%)	[133]
1–22	6-phytase	0–2000	ADG (29%), ADFI (20%), G:F (10%), bone ash (15%), P digestibility (21%), N digestibility (2%), Ca digestibility (13%), phytate digestibility (27%), P retention (25%), N retention (−5%), Ca retention (36%), phytate retention (25%)	[134]
1–43	6-phytase	0–500	Duodenum Ca concentration (−11%), duodenum P concentration (−8%), jejunum P concentration (−9%), serum P (9%), bone ash (4%), bone Ca (7%), bone zinc (24%)	[135]
1–43	6-phytase	0–500	ADG (2%), FCR (−2%), blood glucagon (−21%), blood glucose (6%), blood triglycerides (−17%)	[136]
1–22	6-phytase	0–1500	ADG (10%), ADFI (5%), FCR (−4%), bone ash (4%), gizzard inositol concentration (45%), gizzard inositol hexa-phosphate concentration (−100%), gizzard inositol tri-phosphate concentration (−71%)	[137]
1–43	6-phytase	0–5000	ADG (3%), FCR (−3%), ileum pH (7%), ileum *Lactobacillus* sp. (2%), ileum *Streptococcus/Lactococcus* (−1%), ileum total SCFA ^14^ (22%), ileum DL-lactic acid (15%), ileum acetic acid (34%), crop pH (−1%), crop total SCFA (48%), crop acetic acid (24%)	[13]
1–22	6-phytase	0–2000	ADG (16%), ADFI (10%), FCR (−7%), tibia ash (15%), toe ash (20%), Ca digestibility (21%), P digestibility (43%), threonine digestibility (3%), indispensable amino acid digestibility (3%), Ca retention (28%), P retention (33%), AME retention (5%)	[138]
7–22	6-phytase	0–4000	ADG (8%), ADFI (5%), G:F (4%), jejunum villus height (14%), bone ash (7%), DM digestibility (4%), N digestibility (5%), energy digestibility (3%), P digestibility (35%), Ca retention (11%), P retention (21%)	[139]
1–23	6-phytase	0–1500	ADG (36%), ADFI (16%), G:F (22%), bone ash (25%), P digestibility (31%), P retention (20%)	[140]
1–37	6-phytase	0–1000	Bone ash (10%), bone potassium (−18%), bone sodium (−15%),	[141]
1–36	6-phytase	0–5000	ADG (9%), ADFI (11%), Ca digestibility (8%), P digestibility (17%), plasma Ca (8%), plasma P (18%), P excretion (−32%), bone ash (11%), bone Ca (8%), bone P (10%)	[142]
1–42	6-phytase	0–2000	Final body weight (32%), FCR (14%), ADFI (22%), total amino acid digestibility (6%)	[143]
1–22	6-phytase	0–1500	ADG (14%), FCR (13%), DM digestibility (14%), N digestibility (18%), DM retention (5%), N retention (10%), gizzard inositol (36%), gizzard inositol hexaphosphate (85%), ileum inositol (40%)	[144]
1–42	6-phytase	0–6000	ADFI (9%), FCR (4%), serum Ca (−13%), serum P (34%), serum total protein (16%), Ca digestibility (27%), P digestibility (36%), CP digestibility (6%), bone P (12%),	[145]
1–28	6-phytase	0–2500	Ileum ash (−11%), ileum P (−21%)	[146]
1–42	6-phytase	0–2000	ADG (3%), FCR (−3%)	[147]
2–23	6-phytase	0–1500	ADG (7%), ileum total inositol phosphate (−41%), ileum inositol hexaphosphate (77%)	[148]
1–36	6-phytase	0–1000	ADFI (2%), P digestibility (23%), Ca digestibility (38%)	[149]
1–22	6-phytase	0–4500	ADG (14%), Ca digestibility (−19%), P digestibility (15%), gizzard inositol (59%), gizzard inositol hexaphosphate (−99%), gizzard inositol biphosphate (20%), ileum inositol (56%), ileum inositol hexaphosphate (−91%), ileum inositol triphosphate (71%), sodium/glucose cotransporter 11 (79%)	[150]
5–26	6-phytase	0–1000	ADG (21%), ADFI (15%), CP digestibility (3%), lysine digestibility (2%), P digestibility (20%), Ca retention (22%), P (21%) retention	[86]
1–42	6-phytase	0–3000	ADG (6%), FCR (−2%), carcass fat (7%)	[151]
8–22	6-phytase	0–3000	G:F (1%), plasma *myo*-inositol (37%), duodenum-jejunum inositol hexaphosphate disappearance (83%), duodenum-jejunum Ca disappearance (13%), duodenum-jejunum P disappearance (37%), ileum inositol hexaphosphate disappearance (71%), ileum Ca disappearance (6%), ileum P disappearance (31%), duodenum-jejunum *myo*-inositol (56%), ileum *myo*-inositol (71%), mucin2 gene expression (83%)	[152]
1–35	6-phytase	0–500	AME (2%), N digestibility (6%), P digestibility (16%), magnesium digestibility (76%), N retention (12%), Ca retention (21%), P retention (27%), magnesium retention (62%), sodium retention (27%), jejunum total protein content (−3%), Ca-ATPase activity (10%)	[153]
1–42	6-phytase	0–40,500	ADG (4%), ADFI (4%), FCR (−1%), final body weight (4%), mortality (−69%), carcass yield (1%)	[154]
1–42	6-phytase	0–2500	DM digestibility (7%), ash digestibility (4%), Ca digestibility (4%), P digestibility (6%), plasma *myo*-inositol (9%)	[155]
1–42	6-phytase	0–3000	ADG (4%), FCR (−6%), BBS (12%)	[156]
1–42	6-phytase	0–8000	ADG (4%), FCR (−2%), AME (2%), lysine digestibility (1%), methionine digestibility (2%), threonine digestibility (2%)	[157]
1–11	6-phytase	0–4000	ADG (22%), ADFI (14%), G:F (9%), tibia ash (22%), energy digestibility (4%), N digestibility (7%), P digestibility (47%), Ca digestibility (15%), lysine digestibility (6%), methionine digestibility (5%), threonine digestibility (7%), tryptophan digestibility (7%), arginine digestibility (5%)	[158]
1–28	6-phytase	0–4000	RA ^15^ of *Pelomonas* (−74%), *Helicobacter* (−92%), *Pseudomonas* (−85%), and *Lactobacillus* (56%), CP digestibility (7%), P digestibility (16%), villus height (11%), BBS (14%), bone ash (5%), bone P (5%)	[14]
12–23	6-phytase	0–4000	ADG (20%), ADFI (11%), G:F (11%), bone ash (19%), DM digestibility (4%), energy digestibility (3%), Ca digestibility (30%), P digestibility (49%), lysine digestibility (7%), methionine digestibility (5%), threonine digestibility (12%), tryptophan digestibility (7%), DM retention (2%), Ca retention (55%), P retention (53%)	[159]

* The described percentages of change were in comparison with treatments without phytase supplementation. The described changes were considered significant with *p* < 0.05 and tendency with 0.05 ≤ *p* < 0.10. ** The references were selected from peer-reviewed literature available after 2000. ^1^ Average daily gain. ^2^ Average daily feed intake. ^3^ Calcium. ^4^ Phosphorus. ^5^ Gain to feed ratio. ^6^ Apparent metabolizable energy. ^7^ Feed conversion ratio. ^8^ No data. ^9^ Dry matter. ^10^ Nitrogen. ^11^ Gross energy. ^12^ Crude protein. ^13^ Bone breaking strength. ^14^ Short chain fatty acids. ^15^ Relative abundance.

**Table 2:** A list of studies since 2000 describing effects of 3- and 6-phytase supplemented individually at different levels in diets for nursery pigs.

Duration, Day of Age	Type	Activity, FTU/kg Feed	% Change *	Reference **
42–70	3-phytase	0–1200	ADG ^1^ (14%), G:F ^2^ (8%), inorganic plasma P ^3^ (6%)	[160]
28–63	3-phytase	0–2500	ADG (21%), Ca ^4^ digestibility (20%), P digestibility (60%)	[161]
15–38	3-phytase	0–400	ADG (20%), G:F (12%), bone ash (15%)	[90]
21–56	3-phytase	0–1250	ADG (34%), ADFI ^5^ (19%), G:F (18%)	[162]
18–46	3-phytase	0–500	Stomach phytate hydrolysis (75%), ileum phytate hydrolysis (15%), rectum phytate hydrolysis (15%), bone ash (19%), P digestibility (40%), histidine digestibility (7%)	[163]
18–32	3-phytase	0–500	ADG (15%), plasma P (89%), liver P (6%)	[164]
27–46	3-phytase	0–750	ADFI (8%), plasma zinc, (54%), plasma alkaline phosphatase activity (67%), bone zinc (43%), liver zinc (26%)	[165]
27–46	3-phytase	0–700	ADG (22%), ADFI (14%), G:F (22%), plasma zinc (71%), plasma P (10%), alkaline phosphatase activity (71%), bone ash (9%), bone zinc (48%), bone P (2%)	[166]
31–59	3-phytase	0–1000	ADG (17%), bone ash (6%), Ca digestibility (23%), P digestibility (68%)	[103]
18–46	3-phytase	0–350	ADG (6%), ADFI (6%), G:F (4%), bone ash (4%), bending moment (16%)	[167]
28–70	3-phytase	0–750	ADG (6%), final body weight (5%)	[168]
ND ^6^	6-phytase	0–500	Ca digestibility (13%), P digestibility (40%)	[169]
ND	6-phytase	0–600	ADG (11%), body weight (7%), plasma P (24%)	[170]
18–37	6-phytase	0–500	G:F (1%), no changes in other parameters	[171]
20–34	6-phytase	0–500	Plasma zinc (5%), Ca retention (4%), P retention (3%)	[172]
28–71	6-phytase	0–15,000	ADG (28%), ADFI (22%), G:F (9%), ash digestibility (25%), Ca digestibility (25%), P digestibility (60%)	[173]
30–58	6-phytase	0–12,500	ADG (34%), ADFI (17%), G:F (22%), BBS ^7^ (51%), ash content (47%), P absorption (63%), Ca absorption (46%), N ^8^ absorption (36%)	[174]
42–84	6-phytase	0–2000	Femur strength yield-bending moment (12%), femur strength strain (17%), femur strength maximum bending moment (3%), femur zinc content (17%)	[175]
26–47	6-phytase	0–500	ADG (17%), G:F (11%), CP ^9^ digestibility (9%), P digestibility (16%), bone ash (6%), bone P (4%)	[176]
21–63	6-phytase	0–2500	ADG (2%), final body weight (1%), serum P (5%), serum zinc (60%)	[177]
28–56	6-phytase	0–20,000	ADG (22%), ADFI (14%), G:F (9%), BBS (43%), bone ash weight (28%), DM ^10^ digestibility (4%), GE digestibility (4%), CP digestibility (5%), Ca digestibility (36%), P digestibility (49%), inositol hexaphosphate digestibility (86%), lysine digestibility (5%), threonine digestibility (6%), valine digestibility (5%)	[178]
35–63	6-phytase	0–20,000	ADG (22%), FCR ^11^ (−11%), DM digestibility (2%), GE ^12^ digestibility (2%), CP digestibility (4%), Ca digestibility (37%), P digestibility (52%), BBS (37%), bone ash (32%), bone Ca (35%), plasma P (14%)	[179]
28–48	6-phytase	0–2500	ADG (20%), P fecal digestibility (23%), plasma copper (−21%)	[180]
40–61	6-phytase	0–1000	ADG (15%), ADFI (56%), G:F (10%), bone weight (24%), bone ash (15%)	[181]
21–63	6-phytase	0–4000	ADG (7%), G:F (3%), bone ash (10%)	[182]
28–70	6-phytase	0–2500	ADFI (−9%), FCR (−8%), stomach pH (−20%)	[183]
21–62	6-phytase	0–4000	ADG (21%), ADFI (25%), P digestibility (54%), BBS (28%), bone ash (14%)	[184]
32–41	6-phytase	0–2000	AID ^13^ (27%) and ATTD ^14^ (46%) of P, ATTD of Ca (16%), N digestibility (11%), inositol hexaphosphate digestibility (53%), lysine digestibility (8%), threonine digestibility (8%), valine digestibility (9%)	[185]
21–70	6-phytase	0–2000	ADG (35%), ADFI (17%), G:F (11%), P digestibility (26%), Ca digestibility (14%), Ca absorption (37%), P absorption (32%), plasma *myo*-inositol (39%), duodenum *myo*-inositol (68%), ileum *myo*-inositol (87%), inositol hexaphosphate hydrolysis (98%)	[186]
ND	6-phytase	0–2000	ADG (7%), ADFI (6%), G:F (1%)	[187]
21–42	6-phytase	0–1000	ADG (27%), ADFI (13%), BBS (48%), bone ash (22%), bone Ca (17%), bone P (25%)	[188]
31–59	6-phytase	0–3000	ADG (13%), AID (48%) and ATTD (31%) of P, GLUT2 ^15^ gene expression (55%), ASCT2 ^16^ gene expression (37%), CLDN3 ^17^ gene expression (41%), duodenum-jejunum Inositol hexaphosphate (−92%), ileum inositol hexaphosphate (−95%), duodenum-jejunum *myo*-inositol (67%), ileum *myo*-inositol (80%)	[189]
ND	6-phytase	0–500	ADG (18%), G:F (9%), bone ash (4%)	[190]
35–77	6-phytase	0–4000	ADG (45%), bone mineral density (15%), bone mineral content (17%), fecal *Lachnospiraceae* (ND), fecal *Succinvibrio* (ND), fecal *Bifidobacterium* (ND)	[191]
ND	6-phytase	0–16,000	Stomach pH (−16%), fecal score (−14%), diarrhea frequency (−41%)	[192]
26–68	6-phytase	0–500	ADG (22%), ADFI (15%), Ca digestibility (25%), P digestibility (78%), bone ash (18%)	[193]
21–66	6-phytase	0–5000	ADG (20%), BBS (33%), bone ash (9%), P bone content (12%), CP digestibility (3%), EE ^18^ digestibility (11%), P digestibility (6%)	[84]

* The described effects were in comparison with treatments without phytase supplementation. Described effects were considered significant with *p* < 0.05 and tendency with 0.05 ≤ *p* < 0.10. ** The references were selected from peer-reviewed literature available after 2000. ^1^ Average daily gain. ^2^ Gain to feed ratio. ^3^ Phosphorus. ^4^ Calcium. ^5^ Average daily feed intake. ^6^ No data. ^7^ Bone breaking strength. ^8^ Nitrogen ^9^ Crude protein. ^10^ Dry matter. ^11^ Feed conversion ratio. ^12^ Gross energy. ^13^ Apparent ileal digestibility. ^14^ Apparent total tract digestibility. ^15^ Glucose transporter type 2. ^16^ Neutral amino acid transporter. ^17^ Claudin 3. ^18^ Ether extract.
